# Full-Thickness Skin Graft From an Amputated Part: Review of Successful Treatment of Class III Ring Avulsion Injury at Bedside in the Emergency Department

**DOI:** 10.7759/cureus.14405

**Published:** 2021-04-10

**Authors:** Joseph P Kelly, Benjamin Catoe, David H MacDonald

**Affiliations:** 1 Orthopaedic Surgery Residency, Jack Hughston Memorial Hospital, Phenix City, USA; 2 Orthopaedic Surgery, The Hughston Foundation, Columbus, USA; 3 Orthopaedic Surgery, The Hughston Clinic, Columbus, USA

**Keywords:** amputation, coverage, full-thickness skin graft, hand, ring avulsion, bedside, single stage

## Abstract

Urbaniak class III ring avulsion injuries involve significant soft tissue and bone loss. Management typically focuses on immediate, temporary soft tissue coverage followed by a planned trip to the operating room for either amputation or replantation. While soft tissue coverage is of utmost importance, maintenance of digital length, functionality, and cost-effectiveness of viable treatment options should also be considered. The use of soft tissue from amputated structures is well documented, especially in the case of planned surgical amputations. This method has also been known to be used in the case of hand injuries with severe soft tissue compromise; however, there are no known, documented reports of acute treatment of injuries such as ring avulsions with such methods. In this report, we present a case of a class III ring avulsion injury treated utilizing a single-stage, full-thickness skin graft obtained from an amputated part in the emergency department.

## Introduction

Class III ring avulsion injuries according to the Urbaniak classification are those that result in complete degloving or amputation of the digit [1]. Since the advent of microsurgical techniques, it has become more common to treat such injuries with revision amputation, ray resection, or replantation in the case of amputations distal to the flexor digitorum superficialis (FDS) tendon with intact proximal interphalangeal joint (PIPJ) function [2].

Coverage from amputated donor sites has been reported in rare cases of amputations. Dai et al. reported the successful use of degloved skin from amputated parts in the case of multiple above-knee amputations [3]. Küntscher et al. have also reviewed the use of filet flaps for major defects using the concept of “spare parts” from amputated structures. This method allowed for the avoidance of donor-site morbidity in addition to decreased operative time, all while allowing adequate soft tissue coverage [4]. Full-thickness skin grafts have been harvested from excised supernumerary digits with patients demonstrating 100% acceptance of the graft and full range of motion in grafted fingers [5]. Full-thickness graft coverage has been anecdotally used for acute soft tissue management of degloving injuries of the hand. To the best of our knowledge, however, there are no documented reports of full-thickness grafts procured from amputated donor parts in the acute setting as definitive, single-stage treatment. Here we present a case of such treatment for an active-duty soldier having sustained a Urbaniak class III ring avulsion injury.

## Case presentation

The patient is a 21-year-old right-hand-dominant male who presented to the emergency department one hour following injury to his left hand. He is an active-duty military male with no comorbidities who was running as part of a physical training exercise. During the run, he jumped and attempted to strike a stop sign with his left hand when his wedding band caught on the top of the sign causing a ring avulsion type injury to his left ring finger. His wound was dressed, and the amputated digit was wrapped in a latex glove and placed in a plastic bag of water and ice.

**Figure 1:**
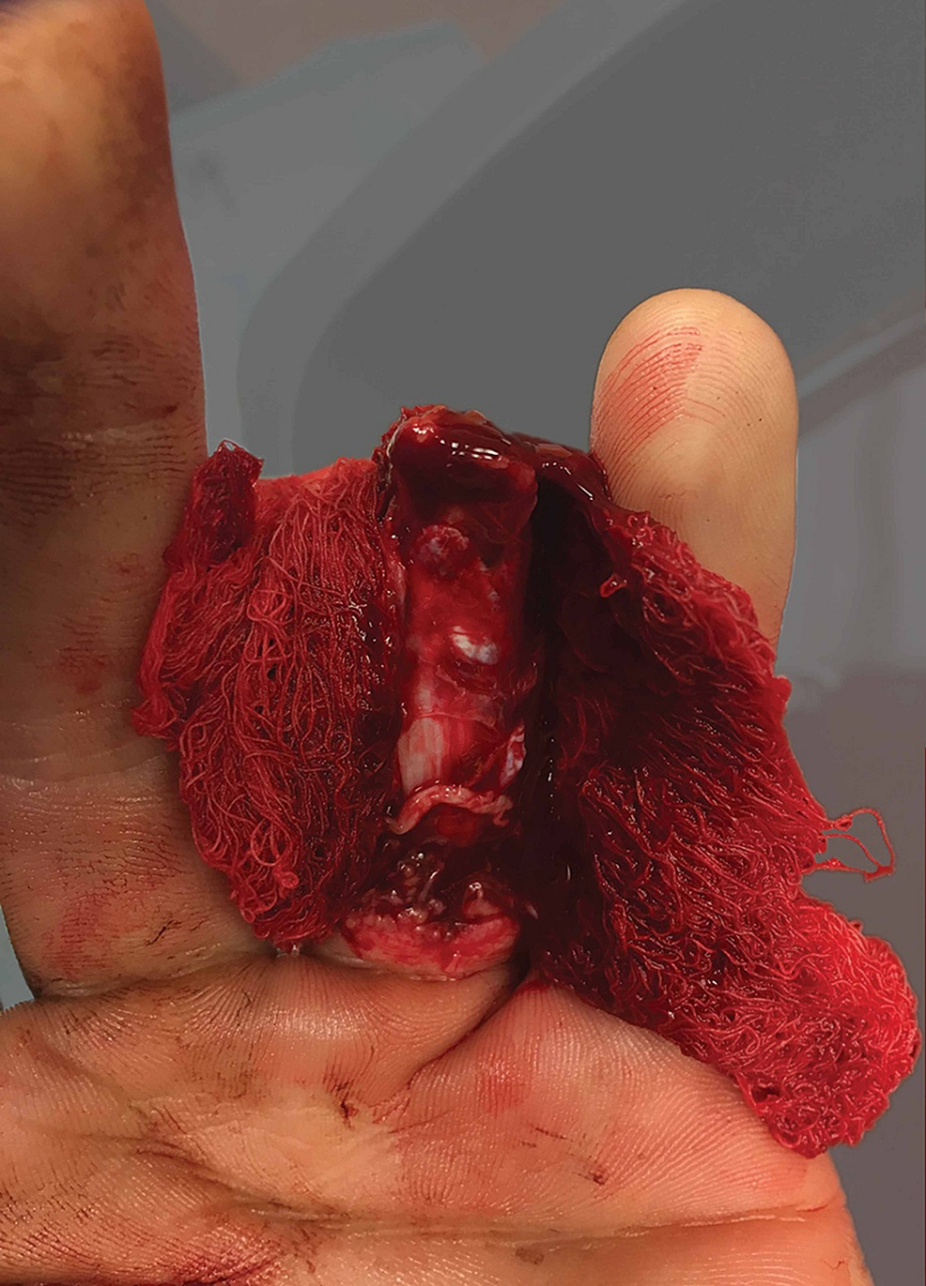
Clinical photo (palmar view) of soft tissue injury.

**Figure 2:**
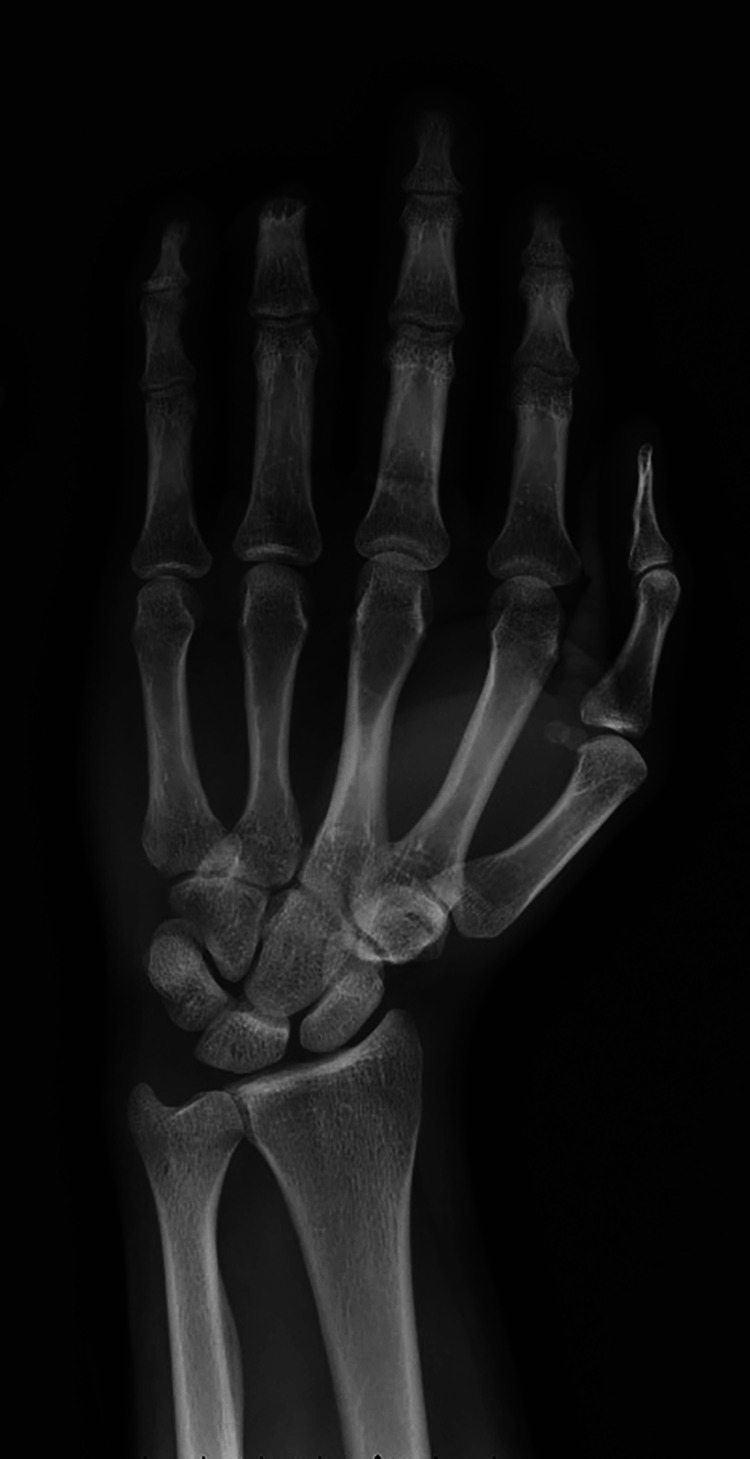
Posteroanterior plain radiograph of the left hand demonstrating amputation through the ring finger middle phalanx.

**Figure 3:**
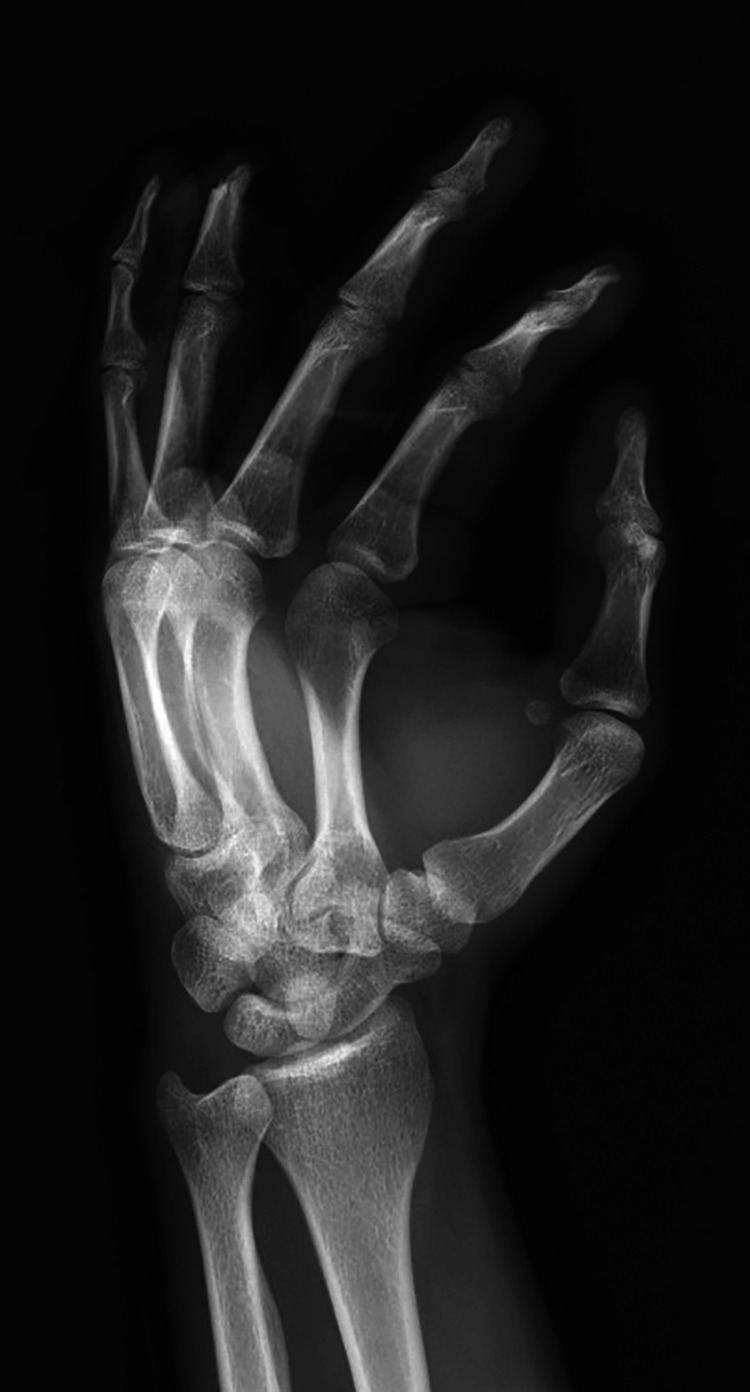
Oblique plain radiograph of the left hand demonstrating amputation through the ring finger middle phalanx.

**Figure 4:**
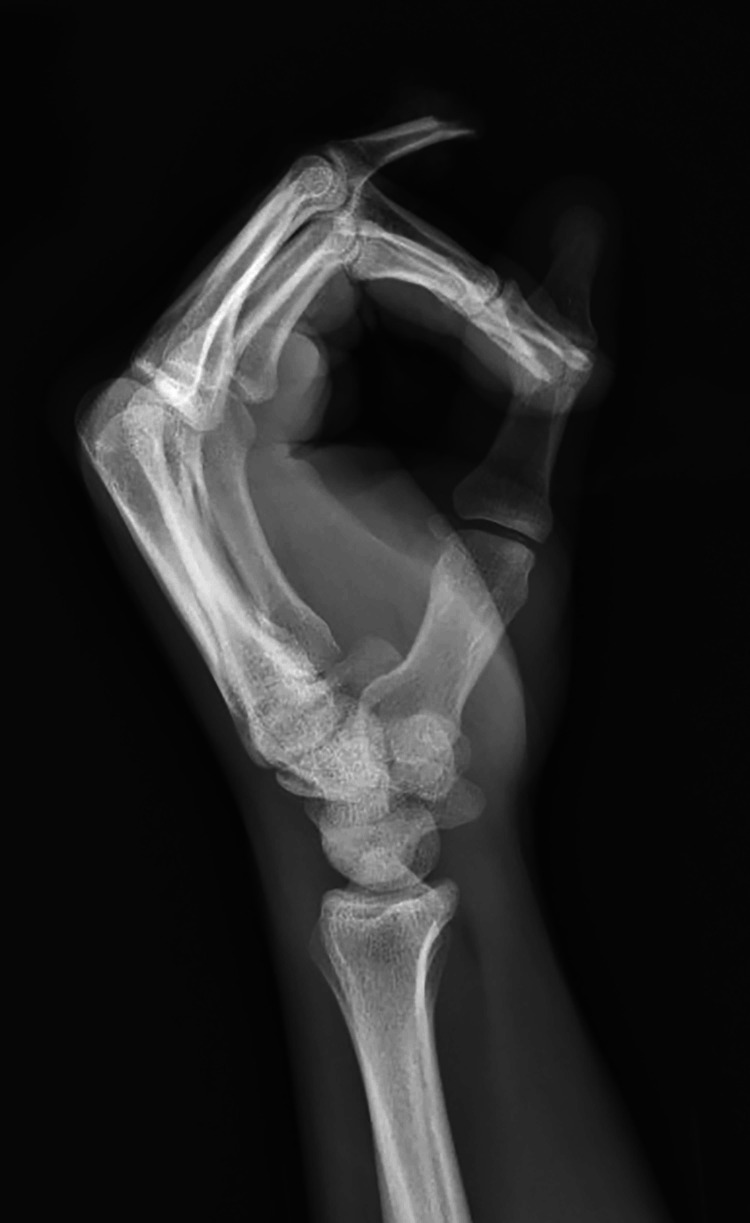
Lateral plain radiograph of the left hand demonstrating amputation through the ring finger middle phalanx.

Upon initial examination, the patient was noted to have sustained a complete amputation of his ring finger at the level of the middle phalanx proximal to the FDS insertion with significant circumferential soft tissue degloving (Figures 1-4).

The patient reported intact sensation to both the radial and ulnar aspects of the finger proximal to the injury, and complete avulsion of the radial and ulnar proper digital neurovascular structures was noted. With the nearest replantation center approximately 100 miles away, a warm ischemia time of 2-3 hours from injury at point of contact, and the significant extent of soft tissue injury, it was determined that the likelihood of successful replantation was low, and revision amputation with primary closure or coverage was recommended to the patient.

Consent was obtained for debridement and primary closure versus graft coverage. Local anesthesia was achieved with a digital block, and a thorough bedside irrigation and sharp debridement were performed under the supervision of the senior staff. The avulsed radial and ulnar arteries were tied off with 4-0 nylon suture. The middle phalanx, which had essentially been skeletonized by the injury, was shortened with a rongeur in an initial attempt to obtain soft tissue coverage. Adequate soft tissue coverage was still not possible despite step-wise shortening of the middle phalanx to the point of complete removal through the PIPJ. The decision was then made to take the extra time to create a full-thickness skin graft from the amputated digit. This would be performed in order to provide adequate soft tissue coverage to the underlying bone and neurovascular structures and increase the likelihood of graft uptake due to the use of autologous tissue, and with the hope of avoiding a future, scheduled trip to the operating room.

**Figure 5:**
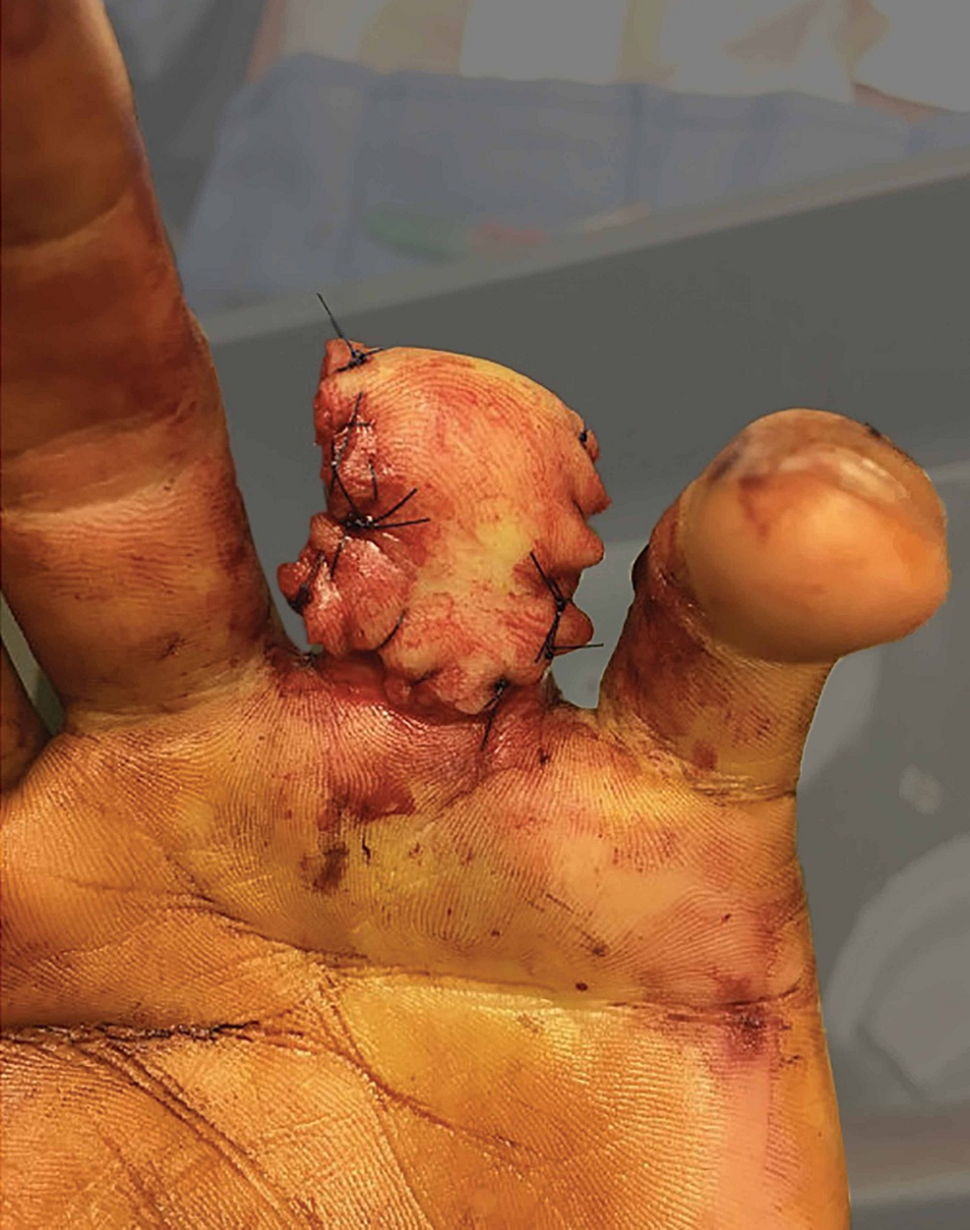
Clinical photo (palmar view) of applied full-thickness skin graft from amputated digit.

**Figure 6:**
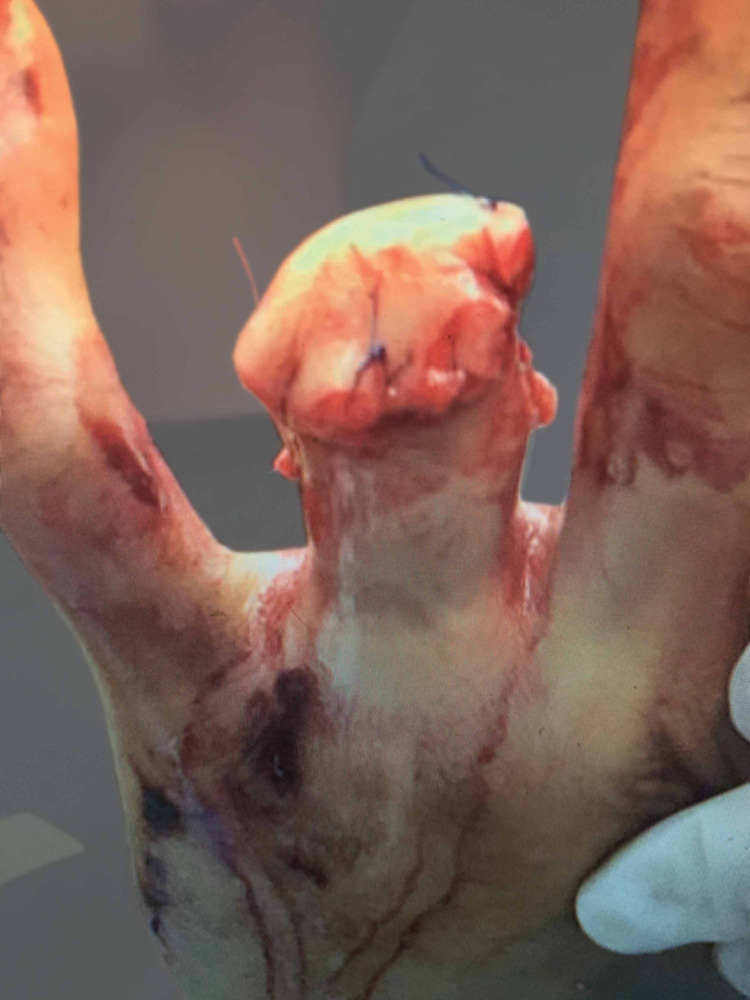
Clinical photo (dorsal view) of applied full-thickness skin graft from amputated digit.

Evaluation of the amputated digit revealed the soft tissue envelope to be intact; the flexor digitorum profundus tendon was also intact and had ruptured proximally from its myotendinous junction in the forearm while still maintaining its insertion at the base of the distal phalanx of the amputated part. The amputated portion of the digit was sterilized in a 10% betadine prep solution. Longitudinal incisions were made on the radial and ulnar sides and connected at the distal tip of the amputated finger to form a fishmouth-type incision. The incision was taken to the bone with a no. 15 blade, and the soft tissue was carefully dissected away from the bone and nail bed. The size of the required graft was then templated and the excess tissue was removed. Subcutaneous fat was excised to the level of the dermis as possible. The graft was secured with 4-0 Prolene suture (Ethicon, Somerville, NJ, USA) in a simple interrupted fashion (Figures 5, 6).

**Figure 7:**
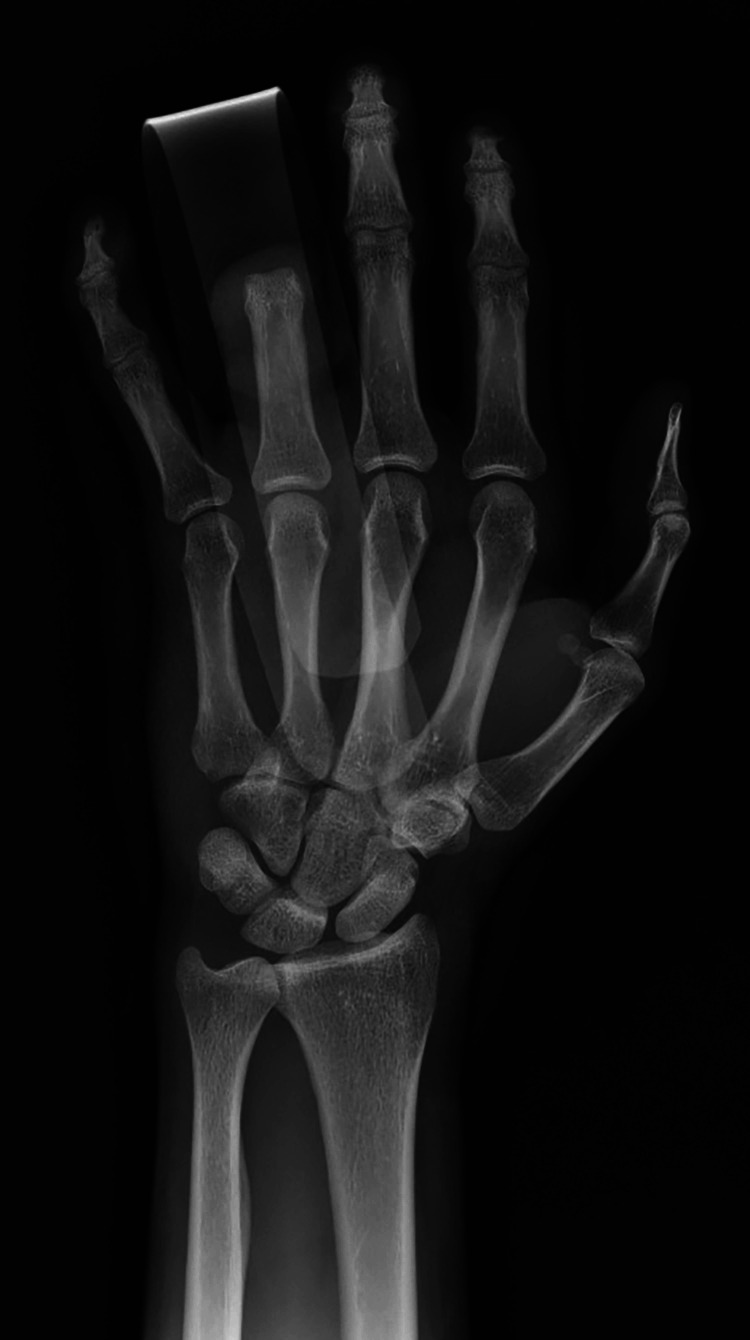
Posteroanterior plain radiograph of the left hand demonstrating revised amputation to the level of the proximal interphalangeal joint post-splint.

A sterile dressing consisting of Adaptic™ non-adherent dressing (3M, St. Paul, MN, USA), 4x4 gauze, and loosely wrapped Kerlix™ (Cardinal Health, Dublin, OH, USA) was applied in addition to an AlumaFoam® splint (Hartmann USA, Inc., Rock Hill, SC, USA) secured with Coban™ wrap (3M) (Figure 7).

The patient was discharged the same day from the emergency department with a prescription for double-strength Bactrim to be taken two times per day for 14 days. He was instructed to follow up in the clinic the following day for wound evaluation.

**Figure 8:**
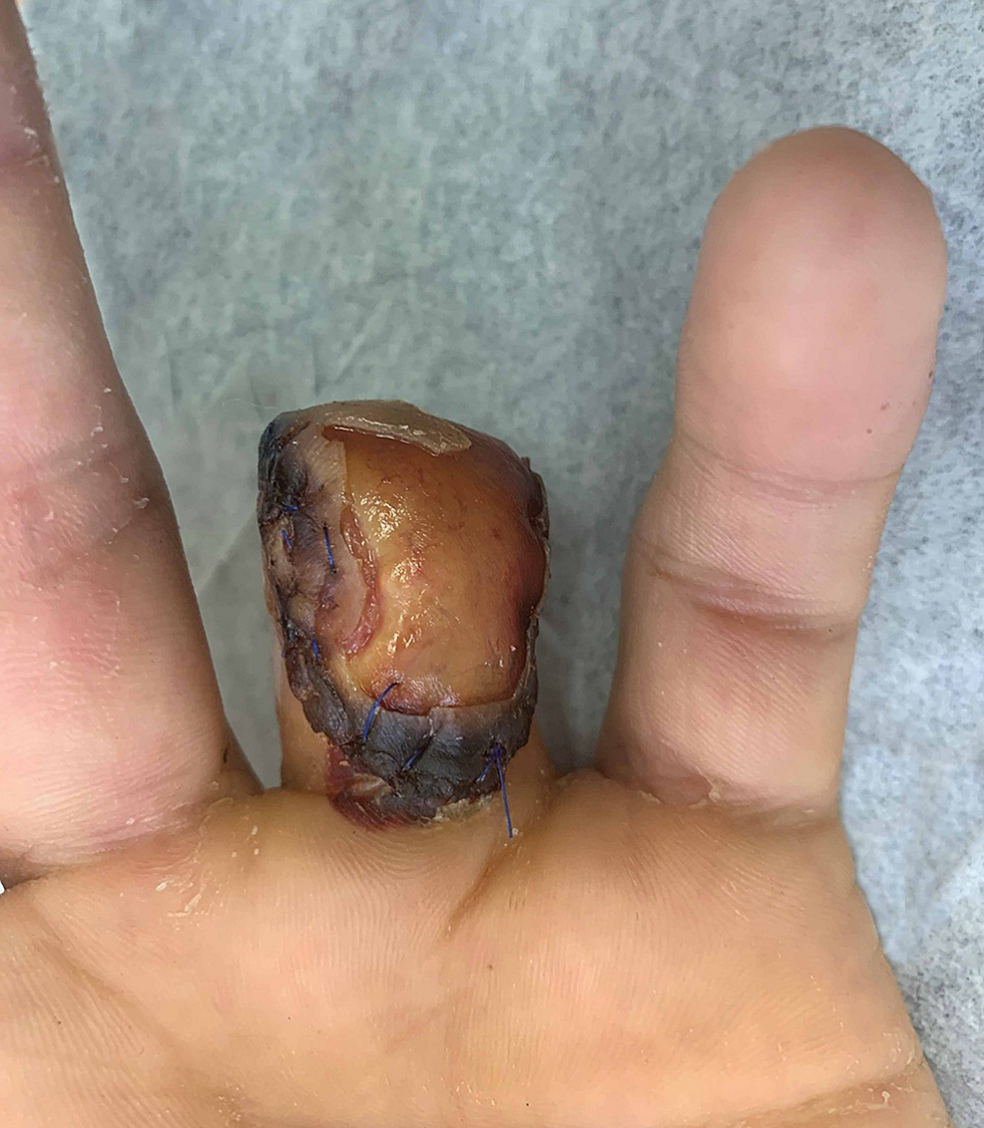
Clinical photo (palmar view) of graft at the six-week follow-up.

**Figure 9:**
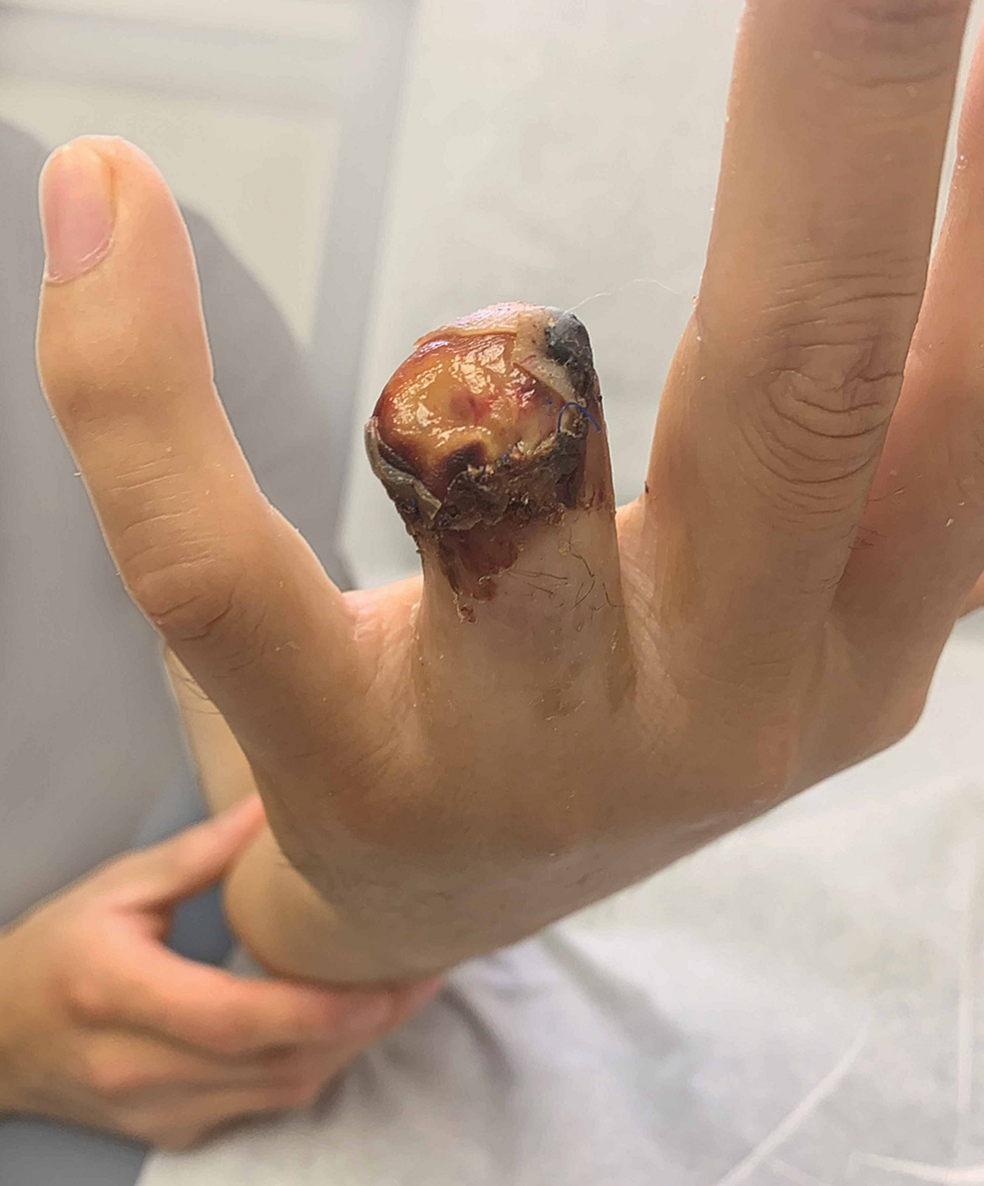
Clinical photo (dorsal view) of graft at the six-week follow-up.

At the initial follow-up, the patient maintained complete coverage of the injury without evidence of necrosis or drainage. He had intact sensation throughout the remaining finger. Nonoperative wound care and monitoring for incorporation of the graft was elected for further treatment. The patient was seen on a weekly basis for the following two weeks with non-weight-bearing restrictions. He continued to demonstrate signs of healing and graft incorporation, with definite signs of central perfusion and only minor ischemia medially and laterally. At his six-week follow-up, he was evaluated for suture removal and was noted to have developed a seroma. His sutures were removed and a small incision was made in the graft in the office to allow for fluid evacuation (Figures 8, 9).

**Figure 10:**
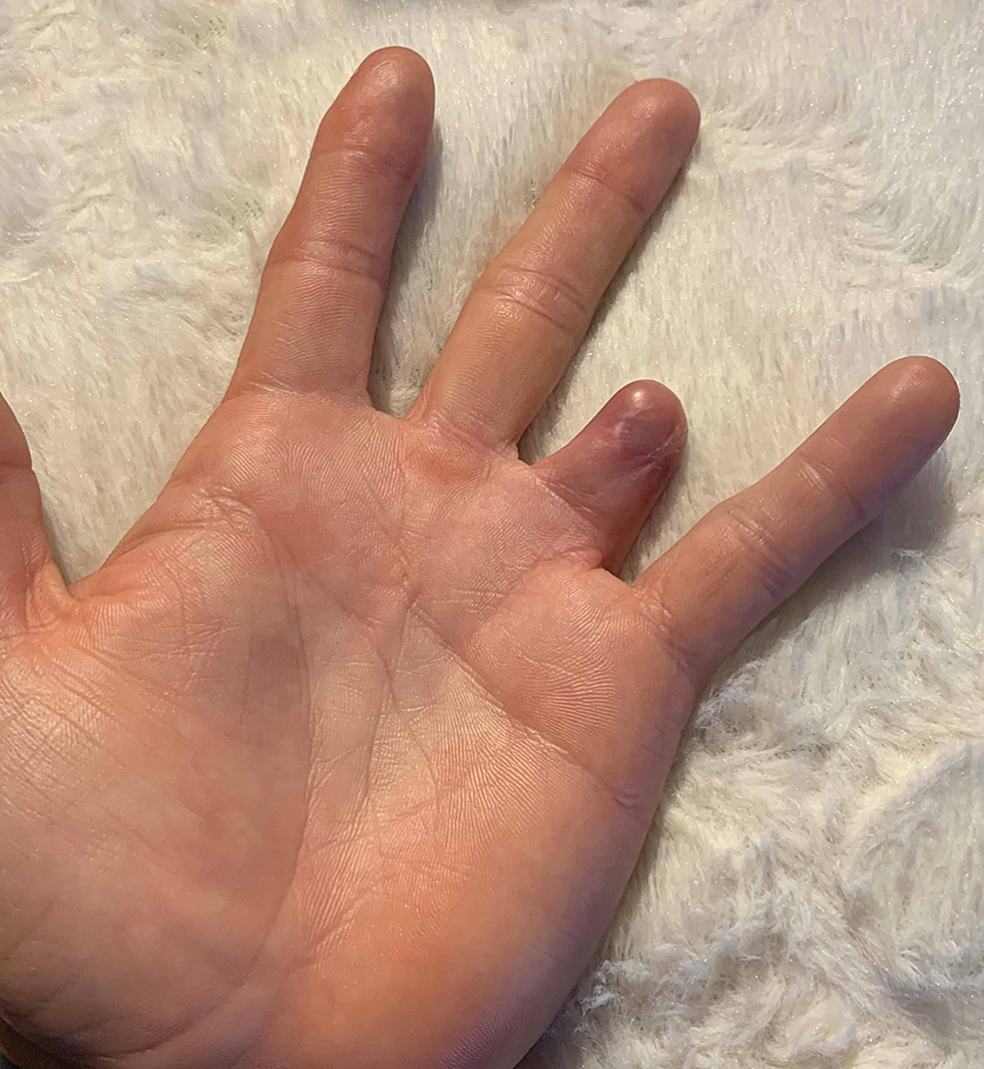
Final clinical photo (palmar view) of the successfully incorporated graft.

**Figure 11:**
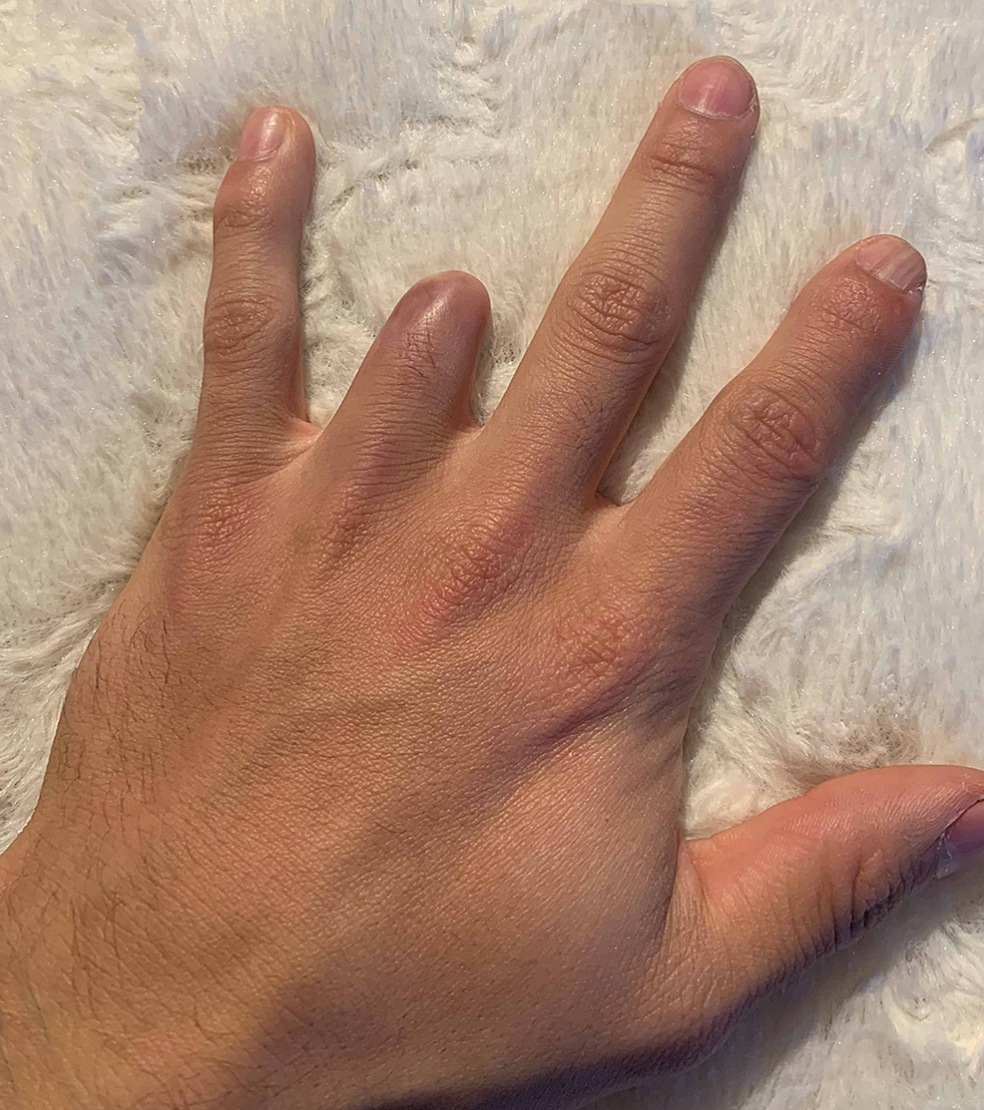
Final clinical photo (dorsal view) of the successfully incorporated graft.

He was allowed to return home with continued wound care and occupational therapy. At the final follow-up, the graft had fully healed with appropriate vascularization and full sensation over the radial and ulnar aspects (Figures 10, 11).

He was cleared to return to full active duty without restrictions.

## Discussion

Class III ring avulsion injuries have historically been treated with replantation, revision amputation, or ray resection depending on the level of amputation in relation to the PIPJ. In this case, a full-thickness skin graft was chosen in order to obtain adequate soft tissue coverage and was used as a definitive treatment over amputation or ray resection due to adequate incorporation without further surgical intervention. Nuzumlali et al. compared results of revision amputation versus ray resection and noted that the residual phalanx may allow for increased stabilization in grip strength and also led to improved patient satisfaction [6]. Sears and Chung reviewed results of replantation for complete finger avulsions and noted protective sensation, but patient-reported outcome scores were actually lower than that of zone II flexor tendon repairs, which historically have had poor results [7]. This kind of procedure, however, has not been thoroughly evaluated in avulsions proximal to the FDS insertion as was the case in our patient. Replantation also requires a facility capable and willing to perform replantation procedures of a single digit that is not a thumb; the closest such facility in this case was located over 100 miles away. Amputated digits are viable for approximately 12 hours of warm ischemia and up to 24 hours of cold ischemia [8]. Full-thickness skin grafts inherently experience immediate post-graft ischemic periods lasting up to 48 hours when placed on a fresh wound, but these grafts can tolerate ischemic intervals between three and five days on a poorly vascularized wound bed [9]. The graft’s ability to tolerate extended ischemia beyond the range of replantation makes it a viable treatment option not only when access to a replantation center is limited but also when presentation to the emergency department may be delayed.

Gil et al. noted similar rates of unplanned secondary revision of fingertip amputations treated initially with primary revision in the emergency department versus those performed in the operating room. Initial management in the emergency department was found to be more cost-effective than in the operating room given these similar rates of secondary revision [10]. While this case only focused on achieving localized soft tissue coverage, we were able to implement the same basic principles and expand upon them by utilizing the viable soft tissue provided from the amputated portion of the digit. Extra time spent at bedside to properly prepare a skin graft from a readily available autogenous site can help avoid donor morbidity and also significantly lower the cost of treatment; in the case of our facility, upwards of $10,000 for operating room and anesthesia time were avoided. While there was a real risk of graft failure, the potential benefit achieved through adequate graft preparation was well worth the extra time in the emergency department. We performed an acceptable and similar procedure in less time, at less cost, and with lower resource utilization compared to what would have been performed in the operating room. We were able to maintain a greater digital length of the digit as compared to revision amputation through the metacarpophalangeal joint or ray resection, potentially allowing him to more effectively complete training and combat tasks required for his profession. The use of amputated parts of the hand in this type of setting can provide a source of soft tissue for permanent treatment and may alleviate the cost and comorbidity associated with more extensive procedures such as amputation, replantation, or microvascular flap coverage.

## Conclusions

Soft tissue from amputated digits can be used successfully for full-thickness grafts in injuries of the hand, such as ring avulsions. This method can be considered for definitive treatment in select patients and serves as an additional treatment option in the armamentarium of the treating physician.

## References

[1] Bamba R, Malhotra G, Bueno RA Jr, Thayer WP, Shack RB (2018). Ring avulsion injuries: a systematic review. Hand (N Y).

[2] Rawles RB, Deal DN (2013). Treatment of the complete ring avulsion injury. J Hand Surg Am.

[3] Dai J, Wang T, Chai Y, Shi H (2016). Full-thickness degloved skin graft provides durable coverage of above-knee amputations with degloving injury of lower extremities. Int J Clinic Exp Med.

[4] Küntscher MV, Erdmann D, Homann HH, Steinau HU, Levin SL, Germann G (2001). The concept of fillet flaps: classification, indications, and analysis of their clinical value. Plast Reconstr Surg.

[5] Rowsell AR, Godfrey AM (1984). A fortuitous donor site for full-thickness skin grafts in the correction of syndactyly. Br J Plast Surg.

[6] Nuzumlali E, Orhun E, Öztürk K, Cepel S, Polatkan S (2003). Results of ray resection and amputation for ring avulsion injuries at the proximal interphalangeal joint. J Hand Surg.

[7] Sears ED, Chung KC (2011). Replantation of finger avulsion injuries: a systematic review of survival and functional outcomes. J Hand Surg Am.

[8] Maricevich M, Carlsen B, Mardini S, Moran S (2011). Upper extremity and digital replantation. Hand (N Y).

[9] Ray S, Rao K (2011). Full thickness skin grafts. Skin Grafts - Indications, Applications and Current Research.

[10] Gil JA, Goodman AD, Harris AP, Li NY, Weiss AC (2020). Cost-effectiveness of initial revision digit amputation performed in the emergency department versus the operating room. Hand (N Y).

